# A case report of cardiogenic shock due to tachycardic atrial fibrillation: a life-saving catheter ablation for atrial fibrillation and prone position during veno-arterial extracorporeal membrane oxygenation

**DOI:** 10.1093/ehjcr/ytaf083

**Published:** 2025-02-18

**Authors:** Masaki Wakita, Yusuke Hosokawa, Shiro Ishihara, Mitsunori Maruyama, Kuniya Asai

**Affiliations:** Department of Cardiology, Intensive Care Unit, Nippon Medical School Musashikosugi Hospital, 1-383 Kosugi-cho, Nakahara-ku, Kawasaki, Kanagawa 211-8533, Japan; Department of Cardiology, Intensive Care Unit, Nippon Medical School Musashikosugi Hospital, 1-383 Kosugi-cho, Nakahara-ku, Kawasaki, Kanagawa 211-8533, Japan; Department of Cardiology, Intensive Care Unit, Nippon Medical School Musashikosugi Hospital, 1-383 Kosugi-cho, Nakahara-ku, Kawasaki, Kanagawa 211-8533, Japan; Department of Cardiology, Intensive Care Unit, Nippon Medical School Musashikosugi Hospital, 1-383 Kosugi-cho, Nakahara-ku, Kawasaki, Kanagawa 211-8533, Japan; Department of Cardiovascular Medicine, Nippon Medical School, 1-1-5, Sendagi, Bunkyo-ku, Tokyo 113-8603, Japan

**Keywords:** Veno-arterial extracorporeal membrane oxygenation, Cardiogenic shock, Prone positioning, Atrial fibrillation, Radiofrequency catheter ablation, Case report

## Abstract

**Background:**

Veno-arterial extracorporeal membrane oxygenation (VA-ECMO) is vital for acute cardio-respiratory failure, but challenges persist sometimes in weaning. Prone positioning, beneficial in veno-venous extracorporeal oxygenation (VV-ECMO), is generally avoided in VA-ECMO patients because of the risks such as bleeding from the catheter insertion site and dislodging of the perfusion cannula or tracheal tube.

**Case summary:**

A 71-year-old male with paroxysmal atrial fibrillation (AF) presented with dyspnoea. Chest computed tomography (CT) and echocardiogram revealed severe pneumonia and decreased cardiac function [left ventricular ejection fraction (LVEF) 20%]. The patient was diagnosed with heart failure complicated by severe pneumonia. Despite treatment, respiratory and circulatory status deteriorated, necessitating a VA-ECMO. Chest CT showed collapsed lungs, especially on the dorsal side. Thus, prone positioning was initiated under VA-ECMO with attention to potential complications associated with the postural change. Then, his respiratory status was improved dramatically. However, the haemodynamic status could not be probably because of tachycardic AF. Electrical cardioversions failed to maintain sinus rhythm, and heart rate was not adequately controlled. We decided to perform radiofrequency catheter ablation of tachycardic AF under VA-ECMO. Pulmonary vein isolation and conduction block line in the cavo-tricuspid isthmus were made and converted to sinus rhythm, enabling weaning of VA-ECMO. The primary cause of the heart failure was considered to be tachycardia-induced cardiomyopathy, as the LVEF improved to 55%.

**Discussion:**

Radiofrequency catheter ablation for AF can be effective even in case of cardio-respiratory failure necessitating VA-ECMO. Furthermore, prone positioning can be performed not only in VV-ECMO but also in VA-ECMO.

**Learning points**

- Prone positioning can be performed not only in cases of veno-venous extracorporeal oxygenation but also under veno-arterial extracorporeal membrane oxygenation (VA-ECMO).
- Radiofrequency catheter ablation for atrial fibrillation can be an effective intervention even in case of cardio-respiratory failure necessitating VA-ECMO support.

## Introduction

Veno-venous extracorporeal membrane oxygenation (VV-ECMO) is a treatment option for patients with acute respiratory distress syndrome (ARDS) refractory to medical therapy.^1^ Conversely, veno-arterial extracorporeal membrane oxygenation (VA-ECMO), a crucial intervention for acute cardio-respiratory failure, is used as extracorporeal cardiopulmonary resuscitation (ECPR) for critical conditions such as lethal ventricular arrhythmias.^2^ Improvement of both circulatory and respiratory status is essential for successful weaning from VA-ECMO, but not sufficient in some patients. While prone positioning has been employed to respiratory support for refractory to medical therapy in VV-ECMO patients, it is generally avoided in VA-ECMO patients because of the associated risks such as bleeding from the catheter insertion site and dislodging of the shorter (∼20 cm) arterial cannula inserted via the femoral artery than the venous cannula.

Herein, we present a patient with tachycardic atrial fibrillation (AF) with cardio-respiratory failure requiring VA-ECMO. Restoration of sinus rhythm by radiofrequency catheter ablation (RFCA) of AF greatly facilitated to wean from VA-ECMO. Furthermore, this case represents prone positioning as an effective adjunctive therapy under VA-ECMO support.

## Summary figure

**Figure:**
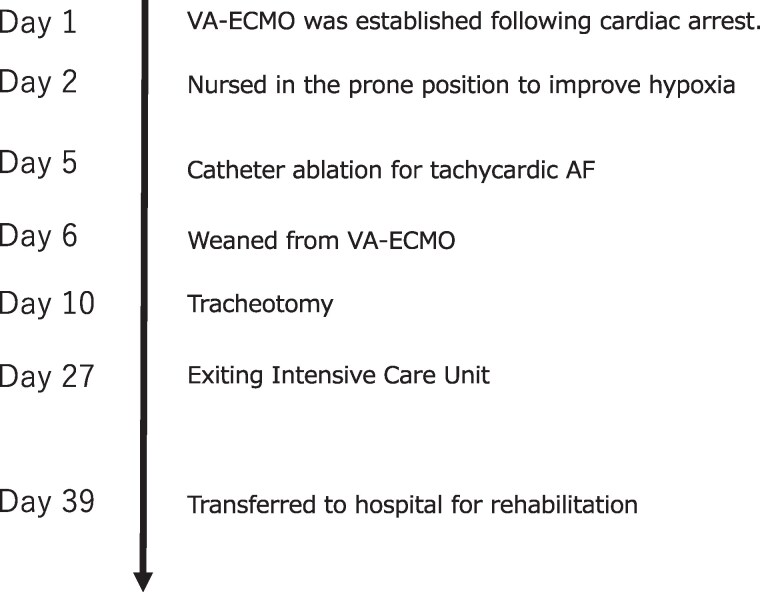


## Case presentation

**Figure 1:**
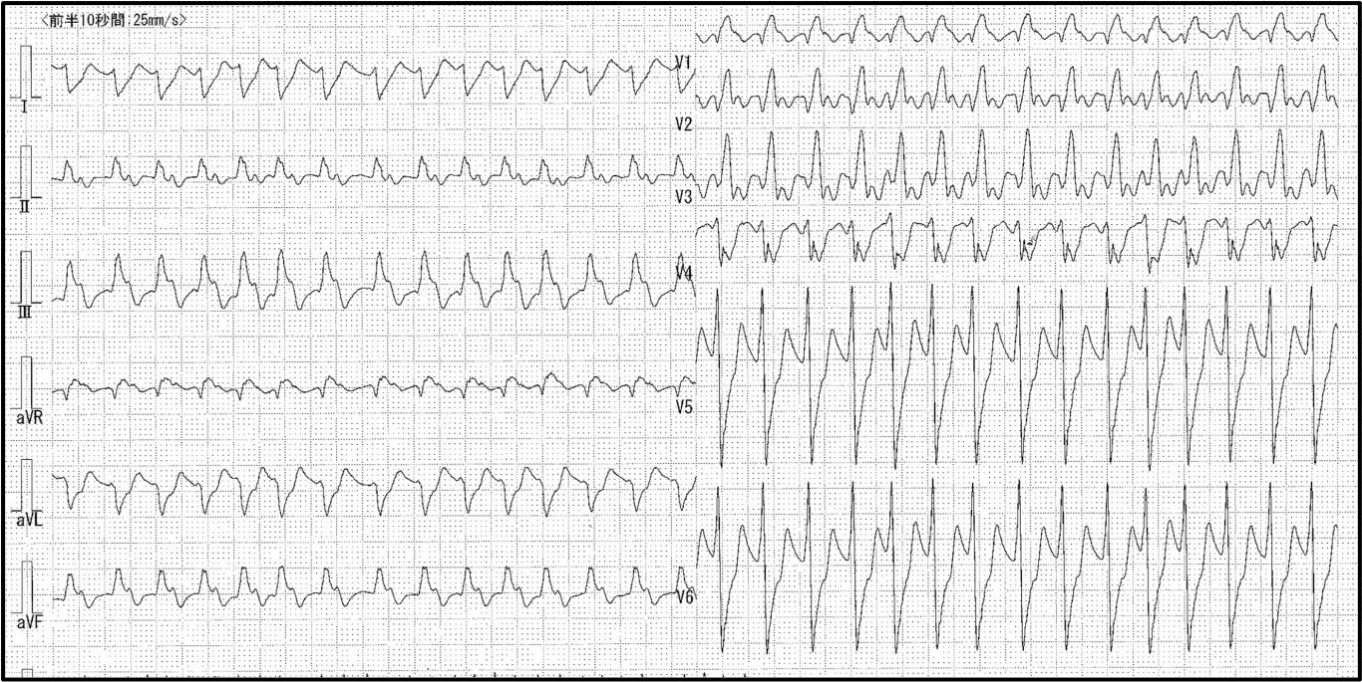
Initial electrocardiogram showed tachycardic atrial fibrillation and ST elevation in aVR lead and ST depression in V3–V6 leads.

A 71-year-old Asian man with a history of hypertension and paroxysmal AF presented to our emergency department with anorexia and dyspnoea. Upon admission, his presentation included a heart rate of 178 b.p.m., a blood pressure reading of 86/40 mmHg, and an oxygen saturation level of 78% (10L reservoir mask). Physical examination demonstrated coarse crackles in both lower lung fields. A 12-lead electrocardiogram showed tachycardic AF with ST elevation in aVR lead and ST depression in V3–V6 leads (*Figure 1*).

**Figure 2:**
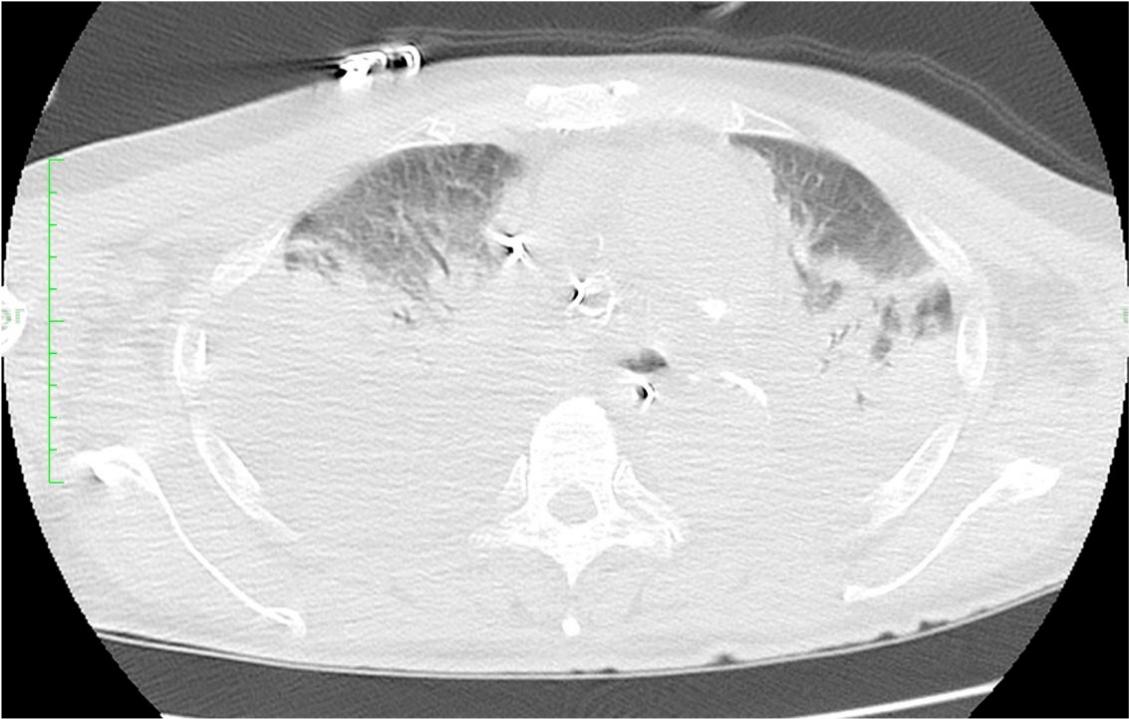
Massive bilateral consolidation was revealed by chest computed tomography.

Blood tests revealed significant abnormalities, including a white cell count of 13 140/μL (reference 4000–9000), C-reactive protein 6.27 mg/L (reference 0–0.3), N-terminal pro-B-type natriuretic peptide 15 494 pg/mL (reference < 125), and troponin T 0.173 ng/mL (reference < 0.014). Chest radiograph and computed tomography (CT) showed bilateral consolidation (*Figure 2*). The echocardiography revealed impaired left ventricular ejection fraction (LVEF) at 20%. The patient was diagnosed with heart failure complicated by severe pneumonia. He developed pulseless electrical activity due to hypoxia and had return of spontaneous circulation within a few minutes by cardiopulmonary resuscitation. However, his respiratory and circulatory status gradually worsened. Thus, we introduced a VA-ECMO via the right femoral artery and vein with a flow of 3.0 L/min, and norepinephrine (0.15 μg/kg/min) was used to maintain stable haemodynamics.

**Figure 3:**
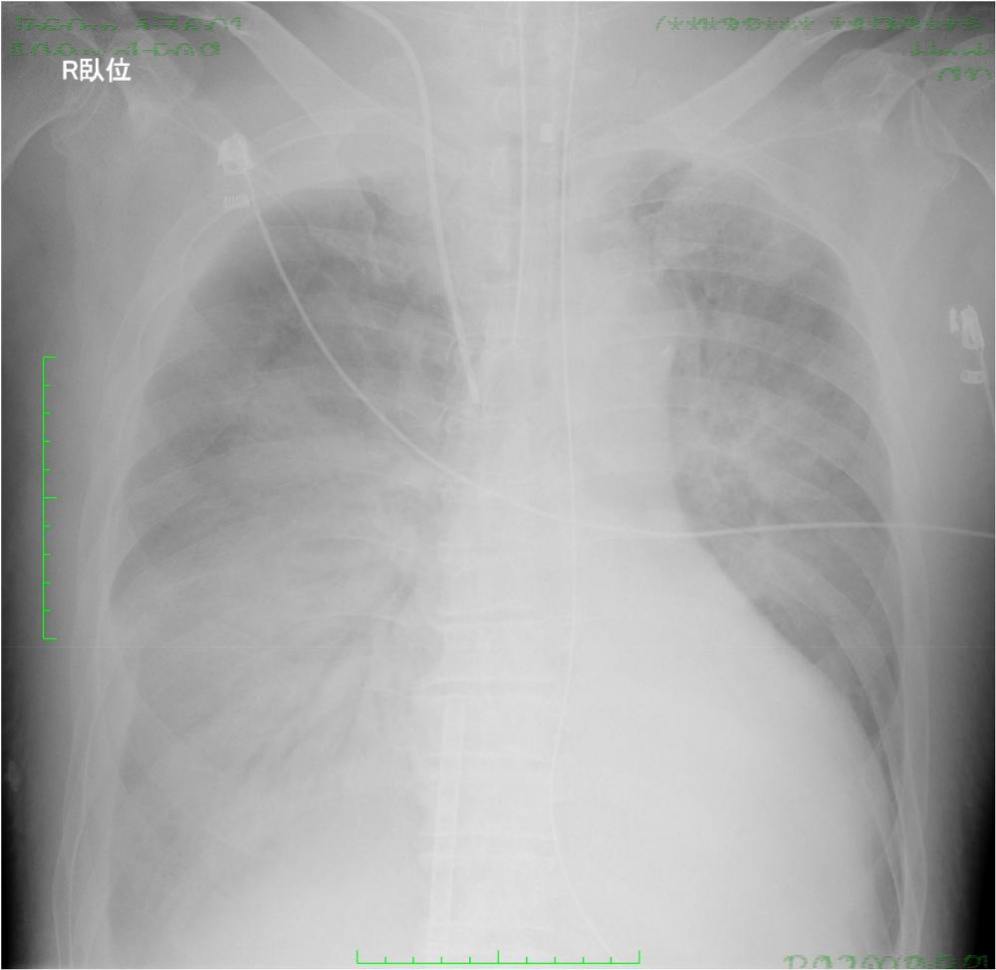
Chest radiograph revealed minimal air content in the bilateral lung fields.

**Figure 4:**
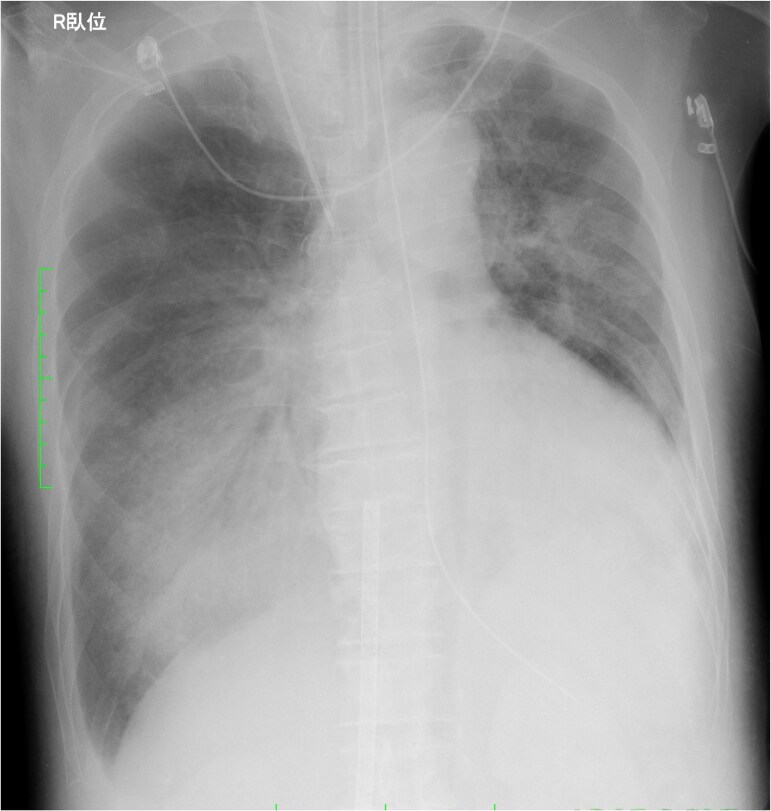
Following prone positioning, chest radiograph findings improved with an increase in air content.

Antibiotic administration and a lung protective ventilation strategy were initiated. Nevertheless, it was difficult of ventilator weaning. Despite maintaining systemic oxygenation with VA-ECMO, there was minimal air content in the bilateral lung fields on chest X-ray (*Figure 3*). Although respiratory physical therapy was performed with adjusting the ventilator setting, chest CT showed collapsed lungs, especially on the dorsal side. Thus, we introduced prone positioning under VA-ECMO to improve respiratory status with attention to potential complications associated with the postural change. Following 8-h prone positioning, his respiratory status and chest X-ray findings (*Figure 4*) improved dramatically with an increase in air content and an increase in P/F ratio from 263 to 910. The settings of ventilator could be reduced from mode PC, PEEP 12 cmH_2_O, peak pressure 34 cmH_2_O, FiO_2_ 80% to mode PC, PEEP 12 cmH_2_O, peak pressure 26 cmH_2_O, and FiO_2_ 60%.

**Figure 5:**
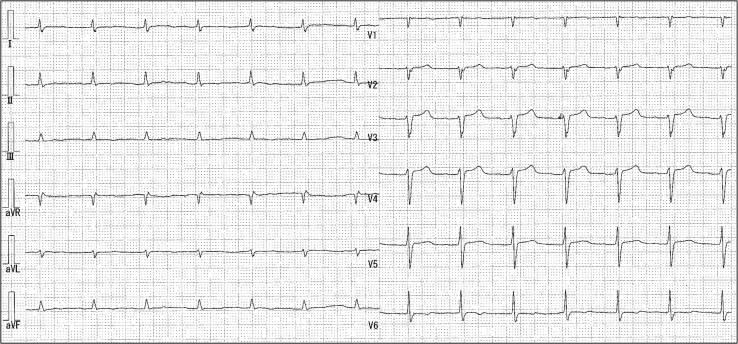
Electrocardiogram after radiofrequency catheter ablation showed sinus rhythm.

While the ventilator settings were successfully reduced, we were not able to stabilize the haemodynamic status probably because of tachycardic AF. Because of coronary angiographic and echocardiographic findings and temporal change of cardiac enzyme, we excluded ischaemic heart disease, myocarditis, or Takotsubo cardiomyopathy. Multiple cardioversions with the intra-venous administration of amiodarone failed to restore sinus rhythm, and heart rate was not adequately controlled pharmacologically. Norepinephrine requirement gradually increased, reaching 0.3 μg/kg/min. Thus, we decided to perform RFCA of tachycardic AF under VA-ECMO on Day 5. A duodecapolar electrode catheter (BeeAT; Japan Lifeline) was inserted into the coronary sinus from the right internal jugular vein. Two long sheaths (Swarts and Agilis; Abbott) were advanced into the left atrium through a single transseptal puncture via the right femoral vein distal to the venous ECMO cannula. A duodecapolar ring catheter (Lasso; Biosense Webster) was used to record the pulmonary vein potentials. Circumferential pulmonary vein isolation and conduction block line in the cavo-tricuspid isthmus were made with an irrigated RF ablation catheter under the guidance of an electroanatomical mapping system (Carto 3; Biosense Webster). Sinus rhythm was successfully restored and maintained after ablation. Using dobutamine (2 μg/kg/min), VA-ECMO flow rate was reduced to 1.8 L/min. The patient was successfully weaned from VA-ECMO on Day 7 with stabilization of his circulatory status in sinus rhythm (*Figure 5*). A tracheostomy was performed on Day 10, considering the need for several weeks to wean the patient off the ventilator. On Day 27, he was weaned from the ventilator and discharged from the Intensive Care Unit to the general ward. The primary cause of the heart failure was considered to be tachycardia-induced cardiomyopathy (TICM), as the LVEF improved to 55%, 28 days after restoration of sinus rhythm. He was then transferred to a rehabilitation hospital on Day 39. After about 5 months of rehabilitation, he had been independently visiting the outpatient clinic. He has continued to attend our hospital since then and has not had any deterioration of heart failure and recurrence of AF.

## Discussion

To the best of our knowledge, this is the first case of TICM treated by RFCA under VA-ECMO, along with prone positioning for ARDS. Tachycardia-induced cardiomyopathy due to persistent tachycardic AF and severe pneumonia worsened the cardio-respiratory status, necessitating the VA-ECMO. Veno-arterial extracorporeal membrane oxygenation is commonly utilized for life-threatening arrhythmias such as electrical storms, ventricular tachycardia, and ventricular fibrillation but is rarely used in TICM patients.^3^ In this case, multiple cardioversions and pharmacological therapy for rhythm and rate control of tachycardic AF failed, and RFCA for AF was performed to restore sinus rhythm and facilitate weaning from VA-ECMO. To date, only one case of RFCA on VA-ECMO for supraventricular tachycardia has been reported, with no previous reports of RFCA for AF during VA-ECMO support.^4^ In certain clinical settings such as ECPR, drainage and perfusion cannulas are typically inserted via the femoral artery and vein, as was done in this patient. Consequently, deciding on the approach for additional interventions, including percutaneous coronary intervention (PCI) or further circulatory support like intra-aortic balloon pumping and Impella, can be challenging in VA-ECMO patients. Novel techniques, such as single-stick access for PCI under VA-ECMO and Impella, have been reported, using a Y-connector into the arterial return cannula.^5^ Normally, RFCA for AF is performed through the right femoral vein. However, we chose to insert the ablation catheter through the left femoral vein first because the ECMO cannula (20 Fr) had been placed through the right femoral vein. Finally, controlling the catheter proved to be challenging, we performed to puncture the right femoral vein distal to the venous cannula for better control. Since procedural complications can indeed occur with AF ablation and could be considered common,^6^ we performed RFCA with caution, focusing on catheter technique and haemodynamic monitoring because of the potentially higher risk in patients on ECMO support.

This patient was placed in prone position for ARDS under VA-ECMO on the second day of illness. The efficacy of prone positioning in ARDS patients is well established,^7^ and it is also performed in patients with VV-ECMO. The guideline for Adult Respiratory Failure from the Extracorporeal Life Support Organization recommends prone positioning for ECMO patients exhibiting consolidation in the posterior lung fields with some anterior lung fields open.^1^ In this case, the CT scan showed air in the anterior lung and consolidation in the dorsal lung, indicating a good candidate for prone position. Conversely, complications associated with prone positioning in ECMO patients include bleeding from the cannula site, which is the most common complication,^8^ the cannula or airway dislodgement, and haemodynamic instability. However, previous studies on the efficacy and complications of prone positioning have primarily included VV-ECMO patients, with no reports available for patients on VA-ECMO. Notably, the arterial cannula for VA-ECMO inserted via the femoral artery is typically shorter (∼20 cm) than the venous cannula. Consequently, prone positioning in VA-ECMO cases carries a higher risk of cannula dislodgement, presenting a potentially hazardous scenario due to patient movement compared with VV-ECMO cases. The guideline also recommends to prevent dislodgement of the ECMO cannulas.^1^ The option of switching to VV-ECMO before prone positioning was unavailable due to haemodynamic instability resulting from tachycardic AF. Therefore, prone positioning was introduced under VA-ECMO, with meticulous attention to cannulas, puncture sites, airway, and haemodynamic, eventually respiratory states resulted in a dramatic improvement.

## Conclusion

Radiofrequency catheter ablation for AF can be an effective intervention even in case of cardio-respiratory failure necessitating VA-ECMO. Additionally, prone positioning can be performed not only in cases of VV-ECMO, but also under VA-ECMO, provided appropriate care is taken.

## Data Availability

The data underlying this article will be shared on reasonable request to the corresponding author.

## Lead author biography

Dr Masaki Wakita has been a fellow in interventional cardiology and critical care cardiology at the Nippon Medical School Musashikosugi Hospital, Kanagawa, Japan since 2020. His main research interests are critical care cardiology (including coronary intervention) and acute heart failure.

## Notes

**Consent:** The authors confirm that written consent for the submission and publication of this case report, including images and associated text, has been obtained from the patient in line with the COPE guidelines.

**Funding:** None declared.
